# Long-Term Associations of Justice Sensitivity, Rejection Sensitivity, and Depressive Symptoms in Children and Adolescents

**DOI:** 10.3389/fpsyg.2017.01446

**Published:** 2017-09-12

**Authors:** Rebecca Bondü, Fidan Sahyazici-Knaak, Günter Esser

**Affiliations:** ^1^Department of Psychology, University of Konstanz Konstanz, Germany; ^2^Department of Psychology, University of Potsdam Potsdam, Germany

**Keywords:** justice sensitivity, rejection sensitivity, depressive symptoms, childhood, adolescence

## Abstract

Depressive symptoms have been related to anxious rejection sensitivity, but little is known about relations with angry rejection sensitivity and justice sensitivity. We measured rejection sensitivity, justice sensitivity, and depressive symptoms in 1,665 9-to-21-year olds at two points of measurement. Participants with high T1 levels of depressive symptoms reported higher anxious and angry rejection sensitivity and higher justice sensitivity than controls at T1 and T2. T1 rejection, but not justice sensitivity predicted T2 depressive symptoms; high victim justice sensitivity, however, added to the stabilization of depressive symptoms. T1 depressive symptoms positively predicted T2 anxious and angry rejection and victim justice sensitivity. Hence, sensitivity toward negative social cues may be cause and consequence of depressive symptoms and requires consideration in cognitive-behavioral treatment of depression.

## Introduction

Depressive symptoms are frequent mental health problems with increasing prevalence during adolescence, particularly among females (Nolen-Hoeksema and Girgus, 1994; Preiß and Remschmidt, 2007; Bertha and Balázs, 2013; Salk et al., 2016). Thus, it is important to identify risk factors for depressive symptoms that are adequate starting points for prevention and intervention in this age range. In late childhood and early adolescence, peer relationships gain importance; the dependence on family relationships remains high (Furman and Buhrmester, 1992). Accordingly, explanation models for depression focussing on interpersonal influences seem particularly significant in this age range (Rudolph et al., 2000; Auerbach and Ho, 2012; Teo et al., 2013). Trait factors reflecting vulnerabilities to negative social cues may then work to intensify the links of negative social experiences and depressive symptoms.

In line with this reasoning, depressive symptoms have been related to *anxious rejection sensitivity* (RS; Ayduk et al., 2001), the tendency to anxiously expect, readily perceive, and inadequately react to alleged rejection (Downey and Feldman, 1996). Angry expectations of rejection—*angry rejection sensitivity—*have been closely related to externalizing problems (London et al., 2007; Zimmer-Gembeck and Nesdale, 2013), whereas relations with depressive symptoms have rarely been examined (Zimmer-Gembeck et al., 2016). This, however, seems important, given that depressive symptoms and aggressive behavior are closely related (Dutton and Karakanta, 2013), particularly in adolescents and girls (Blain-Arcaro and Vaillancourt, 2016). Furthermore, constantly expecting rejection—be it anxiously or angrily—may create stress that can promote depressive symptoms. Thus, our first goal was to examine whether anxious *and* angry RS predict depressive symptoms and/or add to their stability.

Injustice is another social cue individuals may be vulnerable to. People high in *justice sensitivity* (JS) perceive different forms of injustice frequently and show intense negative responses (Schmitt et al., 1995; Schmitt et al., 2010), such as anger, guilt, and rumination (Schmitt et al., 2010) that resemble depressive symptoms. Being unfairly treated may also evoke feelings of worthlessness and helplessness (Bondü et al., in preparation) that are common in depression (American Psychiatric Association [APA], 2013). Thus, cross-sectional research found correlations of JS with depressive symptoms (Bondü and Esser, 2015), but nothing is known about longitudinal links. The second aim of our study, therefore, was to examine whether JS predicts depressive symptoms and/or adds to their stability.

RS and JS may predispose to depressive symptoms by increasing strain, by triggering negative feelings and expectations, and through negative evaluations of social relationships (see below). However, depressive symptoms may also increase the risk of sensible reactions toward social cues, for example when coping requirements are overstrained or when the quality of social relations deteriorates. Accordingly, previous research found anxious RS to be a cause *and* a consequence of depressive symptoms (Marston et al., 2010). Thus, the third aim of our study was to examine whether RS and JS may also be consequences of depressive symptoms.

Finally, girls value social relationships particularly high (Malti and Buchmann, 2010) and should, therefore, be particularly vulnerable to adverse effects of RS and JS. Thus, our last aim was to examine the moderating role of gender in the sensitivity-depression link.

The present study, therefore, seeks to add to the existing knowledge about causes and consequences of depressive symptoms and rejection and justice sensitivity in children and adolescents via latent cross-lagged and latent-change models with two points of measurement. It strives to research the direct links between the concepts and to connect research in personality psychology and developmental psychopathology.

### Depressive Symptoms in Childhood and Adolescence

Childhood and adolescence are critical phases for the development of depressive symptoms (e.g., depressed mood, loss of interest or pleasure, feelings of worthlessness and guilt, thoughts of death; American Psychiatric Association [APA], 2013). In children and adolescents, anger and agitated and aggressive behavior may also signal depression (American Psychiatric Association [APA], 2013). Prevalence rates of major depression are low in childhood (1.9%), but increase to adult level during adolescence (4.5–5%) (Wittchen et al., 1998; Kessler et al., 2003; Preiß and Remschmidt, 2007). Likewise, prevalence rates are similar for girls and boys during childhood, but are twice the size for females than for males from adolescence onward (Angold et al., 1998; Wittchen et al., 1998; Bertha and Balázs, 2013; Salk et al., 2016). Depressive symptoms are highly stable and promote academic and interpersonal problems, suicide risk, and other mental disorders (Fergusson and Woodward, 2002; Naicker et al., 2013). Thus, the early prevention of depressive symptoms is pivotal.

However, little is still known about the genesis of depressive symptoms in children and adolescents. A common idea is the vulnerability-stress perspective, assuming that multiple vulnerability factors may promote depressive symptoms if acute stressors overstrain an individual’s coping resources (Hankin and Abela, 2005; Hamilton et al., 2015). Regarding vulnerability, cognitive factors, such as dysfunctional attitudes or negative cognitive styles, gained much attention, for example in Beck’s Cognitive Model of Depression (Beck, 1975) and Hopelessness Theory (Abramson et al., 1989). These models are well supported in adults, but the evidence for their applicability in children and adolescents is inconsistent (Alloy et al., 2006; Braet et al., 2015). Thus, cognitive aspects alone may be insufficient to explain depressive symptoms in this age range.

Adverse interpersonal experiences in terms of external stressors may, therefore, be particularly important risk factors for depression in adolescence. In line with this reasoning, Rudolph et al. (2000) introduced the Interpersonal Life-Stress Model (Krackow and Rudolph, 2008). According to the model, the interpersonal context is an important source for stress (also see Teo et al., 2013). These stressful experiences may cause individuals to actively add to future stress by developing negative beliefs about the self and relationships, negative attribution styles, and the tendency to focus on negative aspects of interpersonal situations, which may in turn promote depression. Depression, however, may also foster interpersonal stress by promoting interpersonal conflicts or social disengagement that cause existing relationships to deteriorate and hinder new positive relationships to develop (Rudolph et al., 2000, p. 216). Because females put stronger emphasis on social relationships (Malti and Buchmann, 2010), this model also helps to explain common gender differences in prevalence rates of depression.

### Rejection Sensitivity (RS)

RS and JS both reflect inter-individual differences in the perception and interpretation of as well as typical affective responses toward social cues and guide behavior in response to these cues (Bondü and Richter, 2016). They predispose to hypervigilance toward and misinterpretations of ambiguous social cues as negative (Baumert and Schmitt, 2009; Ehrlich et al., 2015). Both may, therefore, help to explain links between depressive symptoms and negative social interactions. Both RS and JS comprise cognitive, but also emotional aspects and, therefore, transcend cognitive factors that have often been related to depressive symptoms so far.

Peer rejection is closely related to depression (Nolan et al., 2003; Slavich et al., 2010). But individuals differ in their tendency to perceive, fear, and suffer from rejection. RS predisposes to anxiously and/or angrily expecting rejection and results from early similar experiences (Downey and Feldman, 1996). Angry RS predisposes to aggressing in response to alleged rejection, whereas anxious RS promotes social withdrawal in the face of perceived rejection (London et al., 2007).

Particularly anxious RS has been linked to depression: It predicted depressive symptoms in women who had been left by their partners (Ayduk et al., 2001) and moderated the link of relationship stressors and depressive symptoms in 16- to 18-year-olds (Chango et al., 2012) even when controlling for initial levels of depression. In 277 adolescents, both anxious (*r* = 0.50) and angry RS (*r* = 0.41) were positively related to and predicted depressive symptoms cross-sectional if considered separately (McDonald et al., 2010). Finally, anxious but not angry RS predicted depressive symptoms in 9- to 14-year olds when controlling for initial levels of depression (Zimmer-Gembeck et al., 2016).

The direction of effect, however, is not clear: In 184 adolescents questioned at 16, 17, and 18 years of age, T1 and T2 anxious RS predicted T2 and T3 depressive symptoms, and T1 and T2 depressive symptoms predicted T2 and T3 anxious RS, indicating a vicious circle of increasing RS and depressive symptoms (Marston et al., 2010). In 331 children, depressive symptoms predicted RS 1 year later, but there was no effect of RS on depressive symptoms (McCarty et al., 2007). Influences on angry RS have not been considered.

### Justice Sensitivity (JS)

Justice-sensitive individuals tend to ruminate. The emotional and behavior response depends on the perspective from which injustice is perceived. Victim-sensitive individuals tend to perceive injustice to their own disadvantage and to respond with anger and the wish to retaliate. Observer-sensitive individuals tend to perceive injustice to the disadvantage of others, to respond with indignation and the wish for victim compensation or perpetrator punishment. Perpetrator-sensitive individuals tend to feel causing injustice and to respond with guilt and the wish for victim compensation or self-punishment (Schmitt et al., 2005, 2010). Victim JS has also been linked to sadness, helplessness, fear of future victimization, and social withdrawal; observer JS was linked to sadness, disappointment, and fear of future victimization (Bondü et al., in preparation).

Higher victim and lower perpetrator JS have been related to externalizing problem behavior (e.g., aggression, ADHD; Bondü and Elsner, 2015; Bondü and Esser, 2015; Bondü and Krahé, 2015). Less is known about the links of JS with internalizing problems such as depression: In adults, the frequency of the perception of injustice to one’s disadvantage and the rumination about these perceptions were positively related to depressive traits (Lovaš and Wolt, 2002). Furthermore, victim JS mediated the link between ADHD and depressive symptoms in children and adolescents cross-sectional (Bondü and Esser, 2015). Finally, victim, observer, and perpetrator JS were positively related to emotional problems including depressive, anxiety, and somatic symptoms in children and adolescents (Bondü and Elsner, 2015) and to neuroticism in adults (Schmitt et al., 2005). Hence, there is some evidence for a relation of JS and depressive symptoms, but nothing is known about mutual long-term effects.

### Links of Rejection and Justice Sensitivity with Depressive Symptoms

Overly negative interpretations of social cues as associated with RS and JS may result in a self-perpetuating circle of adverse responses toward others’ behavior, negative social interactions, and—finally—feelings of loneliness, despair, or hopelessness as associated with depression (Zimmer-Gembeck et al., 2014) and as proposed by the Interpersonal Life-Stress Model (Rudolph et al., 2000). Thus, RS and JS may represent vulnerability factors and increase the risk for social stress through negative interactions at the same time (Hankin and Abramson, 2001; Hamilton et al., 2015). Therefore, both RS and JS should also work to stabilize depressive symptoms if present.

Negative emotions and cognitions associated with RS and JS (Schmitt et al., 1995; Downey and Feldman, 1996) should predispose to emotional problems such as depressive symptoms irrespective of the perspective from which injustice is perceived or whether rejection is anxiously or angrily expected. Because anxious RS is closely related to sadness as the central affect in depression, it should show closer relationships to depressive symptoms than angry RS. However, given that anger and aggression are also linked to depression in childhood and adolescence, angry RS should predict depressive symptoms as well (London et al., 2007; Dutton and Karakanta, 2013; Blain-Arcaro and Vaillancourt, 2016). Repeated perceptions of negative treatment resulting from victim JS and helplessness arising from seemingly unavoidable negative experiences (Bondü et al., in preparation) should work to impair self-esteem. Feelings of guilt as associated with perpetrator JS (Schmitt et al., 2010) should lead to negative perceptions of the self. Hence, victim and perpetrator JS should promote negative self-evaluations in line with Beck’s cognitive triad (Beck, 1975; Braet et al., 2015). Observer JS should predispose to perceiving the world as a bad place, also part of Beck’s cognitive triad (Beck, 1975), as well as to helplessness, the central component of Seligman’s (1972) learned helplessness theory of depression.

Finally, following the reasoning of the Interpersonal Life-Stress Model (Rudolph et al., 2000), depressive symptoms may impair social relationships in the long run (Teo et al., 2013). Frequent negative social experiences can strengthen expectations and perceptions of further negative social interactions, feelings of not being amiable (Ayduk et al., 2001) and, thereby, RS and JS. Given that depressive individuals tend to perceive themselves as victims, victim JS should increase in the long run. A negative view of the world and the self, which is part of the cognitive triad (Beck, 1975) should lead to higher observer and perpetrator JS in the long run. For the same reasons, both anxious and angry RS should increase in individuals with depressive symptoms as well.

### The Present Study and Hypotheses

The present study examined the mutual long-term influences of depressive symptoms and RS and JS as well as the stability of depressive symptoms in *N* = 1,665 children and adolescents from Germany via latent cross-lagged and latent-change models. Our study adds to the present knowledge on depression, RS, JS, and their relations by (a) considering angry RS in a cross-lagged design, (b) linking JS to depressive symptoms as an example for internalizing problem behavior and a frequent mental health problem, (c) considering mutual long-term influences, and (d) examining the moderating roles of gender and age. Thus, our study adds to a better understanding of the relations between developmental psychopathology and sensitivity-related traits. From the theoretical considerations and previous research results outlined above we derived the following hypotheses:

(1) Participants with above average depressive symptoms show higher anxious and angry RS as well as victim, observer, and perpetrator JS than controls at T1 and T2.
(2) RS and JS may add to the genesis of depressive symptoms via a number of pathways. Thus, (a) higher T1 anxious and angry RS and (b) higher T1 victim, observer, and perpetrator JS predict more depressive symptoms at T2: The more sensitive participants are to rejection and injustice at T1, the more depressive symptoms they report at T2.
(3) Depressive symptoms increase a vulnerability to negative social cues. Thus, more depressive symptoms at T1 should predict (a) higher anxious and angry RS and (b) higher victim, observer, and perpetrator JS at T2: The more depressive symptoms a person reports at T1, the more sensitive he/she will be toward rejection and injustice at T2.
(4) RS and JS can be considered continuous vulnerabilities and stressors that should be particularly influential if a person already displays depressive symptoms. Thus, RS and JS should add to the stabilization of depressive symptoms between T1 and T2.
(5) Girls value social relationships particularly high. Thus, links of RS and JS with depressive symptoms should be more pronounced in girls.
(6) Social relationships are important throughout the life-span. Thus, links of RS and JS with depressive symptoms should not differ between children and adolescents.

## Materials and Methods

### Sample

The sample consisted of *N* = 1,665 participants from a larger scale study. They were questioned twice 1–2 years apart. At T1, 1,489 children and adolescents with a mean age of *M* = 13.39 years (*SD* = 2.00; range: 9–19 years; 49.6% girls) participated. At T2, there were 1,299 participants with a mean age of *M* = 14.90 years (*SD* = 1.93; range: 11–21 years; 50.0% female). A total of 1,123 participants took part in both data-waves.

### Material

#### Justice Sensitivity

We measured victim, observer, and perpetrator JS at T1 and T2 with 5 congruently worded items per perspective with the 5-item short version (Bondü and Elsner, 2015) of the Justice Sensitivity Inventory (Schmitt et al., 1995, 2005, 2010; victim JS: “It makes me angry when I am treated worse than others”; observer JS: “I am upset when someone is…”; perpetrator JS: “I feel guilty when I treat someone…”). The items capture the emotional reaction associated with the perception of injustice from the respective perspective, the perceived strain, and the intrusiveness of thoughts. Response options ranged from 0 *totally disagree* to 5 *totally agree.* We computed mean scores for each perspective (range: 0–5). Both the original and the 5-item measure have been shown to be reliable and valid (Schmitt et al., 2010; Bondü and Elsner, 2015).

#### Rejection Sensitivity

We measured RS at T1 and T2 with a translated, short version of the Child Rejection Sensitivity Questionnaire (CRSQ; Downey et al., 1998). We presented participants with five situations possibly resulting in rejection. They indicated how anxious and angry they would feel (1 *not nervous/angry* to 6 *very, very nervous/angry*) and how likely rejection would be (1 *very unlikely* to 6 *very likely*). We computed anxious and angry RS scores by adding the multiplied degree of anger/anxiety and likelihood of rejection per situation and dividing by five (range: 1–30; Downey and Feldman, 1996). The original questionnaire has been shown to be reliable and valid (Downey and Feldman, 1996).

#### Depressive Symptoms

We measured depressive symptoms with 39 dichotomous items (0 *no*, 1 *yes*) from the dysphoria (“Do you cry often?”; 25 items) and psychosomatic complaints (“Do you often feel ill?”; 14 items) subscales of the Deutscher Depressionstest für Kinder (Rossmann, 2005) that has been shown to be reliable and valid (Rossmann, 2005).

### Procedure

We administered all measures during a 1.5–2 h interview in the following sequence: RS, JS, depressive symptoms. Instructions and RS items were read aloud, participants answered the items in silence (computer or paper–pencil). If low reading skill prevented this proceeding, the JS items were also read aloud to the participants. However, in line with instructions, all participants answered the items of the depression questionnaire alone. Participants were guaranteed privacy, parents’ or participants’ informed written consent was collected for all participants in the study. All measures were approved of by the Ethics Committee of the University of Potsdam and the Ministry for Education Brandenburg, Germany.

### Statistical Analyses

We considered participants who displayed 16 or more symptoms of the dysphoria scale (+1.5 *SD*; Butler et al., 1980 for similar proceedings) as showing above average depressive symptoms. That way, 49 (3.3%) participants had above average depressive symptoms at T1 and 37 (2.9%) participants at T2. Participants considered to report above average depressive symptoms did not differ from those in the control group regarding time gap between testing or level of parental education. We therefore did not further consider these two variables in our analyses. These rates resemble point-prevalences of depression among children and adolescents in Germany reported by previous research (Esser et al., 1990; Ihle et al., 2000). Stability, however, was low. Out of the 49 participants who had shown above average depressive symptoms at T1, 33 also participated in T2 (69%). Among these, only 12 still showed above average depressive symptoms (36%). Drop-out analysis revealed a significant effect of the group [MANOVA: *F*(8,1468) = 13.29, *p* < 0.001, $\eta_{p}^{2}$ = 0.07]. On subscale level there were no differences between participants dropping out of and remaining in our study regarding gender, victim and observer JS, angry and anxious RS, and—most importantly—initial levels of depressive symptoms (*p* = 0.596) at T1. However, participants remaining in the study were significantly younger (remaining: *M* = 13.14 years, *SD* = 1.13; drop out: *M* = 14.17, *SD* = 2.00; *p* < 0.001) and showed significantly higher levels of perpetrator JS (remaining: *M* = 3.42 years, *SD* = 1.24; drop out: *M* = 3.11, *SD* = 1.37; *p* < 0.001) than those dropping out.

Given common age differences in depressive symptoms, we divided our sample into two age-groups for some analyses: We considered participants younger than 14 years at T1 as children (*N* = 923, *M* = 12.08 years, *SD* = 1.09, 48.1% girls) and participants 14 years or older as adolescents (*N* = 549, *M* = 15.46 years, *SD* = 1.02; 52.3% girls).

## Results

### Descriptives

**Table 1** shows the internal consistencies, mean values, and standard deviations of all measures in our study for the total group, separately for boys and girls, and separately for participants with elevated depressive symptoms and controls. In the total group, depressive symptoms decreased significantly between T1 and T2 (*p* = 0.001) and anxious as well as angry RS increased significantly between T1 and T2 (*p* = 0.001, respectively; **Table 1**).

**Table 1:** Descriptive statistics for the total sample, separately for boys and girls, and participants high and low in depressive symptoms.

Scale	α	Total sample *M* (*SD*)	Boys *M* (*SD*)	Girls *M* (*SD*)	Depressive group *M* (*SD*)	Control group *M* (*SD*)	*F*	*p^+^*	$\eta_{p}^{2}$
DTK – Depressive Symptoms T1	0.89	8.03 (6.50)^1^	6.83 (5.70)^a^	9.23 (7.02)^b^					
JS – Victim T1	0.79	2.69 (1.14)	2.67 (1.17)	2.80 (1.07)	3.38 (1.07)	2.67 (1.13)	18.412	<0.001	0.012
JS – Observer T1	0.86	2.91 (1.17)	2.69 (1.21)^a^	3.15 (1.04)^b^	3.31 (0.89)	2.90 (1.17)	3.475	0.062	
JS – Perpetrator T1	0.88	3.36 (1.28)	3.07 (1.31)^a^	3.66 (1.14)^b^	3.42 (1.13)	3.36 (1.28)	0.121	0.728	
RS – anxious T1	0.60	7.74 (3.46)^1^	7.36 (3.41)^a^	8.11 (3.46)^b^	10.21 (4.04)	7.63 (3.37)	24.557	<0.001	0.017
RS – angry T1	0.69	4.38 (2.62)^1^	4.52 (2.67)	4.24 (2.56)	5.56 (2.91)	4.31 (2.56)	12.862	<0.001	0.009
DTK – Depressive Symptoms T2	0.90	7.24 (6.40)^2^	5.42 (4.73)^a^	9.08 (7.27)^b^					
JS – Victim T2	0.80	2.74 (1.07)	2.64 (1.08)^a^	2.83 (1.06)^b^	3.29 (0.95)	2.72 (1.08)	7.953	0.005	0.006
JS – Observer T2	0.88	2.89 (1.13)	2.62 (1.14)^a^	3.16 (1.06)^b^	3.38 (1.12)	2.88 (1.13)	2.746	0.098	
JS – Perpetrator T2	0.92	3.26 (1.29)	2.91 (1.33)^a^	3.60 (1.14)^b^	3.51 (1.20)	3.25 (1.29)	0.005	0.946	
RS – anxious T2	0.64	8.09 (3.23)^2^	6.82 (3.14)^a^	7.97 (3.36)^b^	10.79 (4.70)	7.31 (3.19)	32.663	<0.001	0.025
RS – angry T2	0.68	4.80 (2.32)^2^	3.99 (2.23)	3.74 (2.14)	5.11 (2.87)	3.84 (2.16)	14.053	<0.001	0.011


*MANCOVA comparing boys and girls: *n*_*boys*_ = 541, *n*_*girls*_ = 547, superscripts ^a,b^*p* < 0.001; *n*_*depressivet1/t2*_ = 49/36, *n*_*control*_ = 1415/1245; ^+^two-tailed. Superscripts ^1,2^*p* < 0.001 differences between accordant T1 and T2 measures in the total sample.*

A Multiple Analysis of Covariance (MANCOVA) including all measures and controlling for age revealed a significant main effect of gender [*F*(12,1074) = 25.25, *p* < 0.001, $\eta_{p}^{2}$ = 0.22; *n*_boys_ = 541, *n*_girls_ = 547]. Follow-up analyses using ANCOVAs showed that girls reported more depressive symptoms, higher observer JS, perpetrator JS, and anxious RS at T1 and T2 (*p* < 0.001 respectively), as well as higher victim JS at T2 (*p* = 0.001; **Table 1**). A second MANCOVA controlling for gender revealed a significant main effect of the age group [*F*(12,1074) = 25.25, *p* < 0.001, $\eta_{p}^{2}$ = 0.12; *n*_children_ = 739, *n*_adolescents_ = 349]. According to follow-up analyses using ANCOVAs children showed higher anxious RS at T1 and higher angry RS at T1 and T2 (*p* < 0.001 respectively), lower victim JS at T1 and T2 (*p* < 0.001, respectively), and lower observer JS (*p* = 0.006) and depressive symptoms (*p* = 0.049) at T2.

Both at T1 and T2, depressive symptoms were positively correlated with all T1 sensitivity measures and all T2 sensitivity measures but perpetrator JS (**Table 2**).

**Table 2:** Zero-order correlations of depressive symptoms, justice sensitivity, rejection sensitivity, and age for the total sample.

		2	3	4	5	6	7	8	9	10	11	12	13
1	Depressive symptoms T1	0.26***	0.17***	0.07*	0.32***	0.22***	0.60***	0.22***	0.08*	0.00	0.27***	0.17***	0.07**
2	JS-Victim T1	–	0.47***	0.23***	0.19***	0.15***	0.20***	0.43***	0.19***	0.09**	0.11***	0.08**	0.22***
3	JS-Observer T1		–	0.60***	0.09***	-0.01	0.16***	0.21**	0.43***	0.35***	0.08*	0.02	0.06*
4	JS-Perpetrator T1			–	0.06*	-0.07**	0.09**	0.12***	0.35***	0.45***	0.04	-0.04	-0.03
5	RS-anxious T1				–	0.62***	0.20***	0.09**	0.05	0.02	0.39***	0.25***	-0.09***
6	RS-angry T1					–	0.11***	0.08**	-0.01	-0.06	0.24***	0.40***	-0.14***
7	Depressive Symptoms T2						–	0.32***	0.16***	0.05	0.38***	0.21***	0.10***
8	JS-Victim T2							–	0.41***	0.20***	0.23***	0.15***	0.21***
9	JS-Observer T2								–	0.58***	0.10***	-0.03	0.10***
10	JS-Perpetrator T2									–	0.07*	-0.09***	0.03
11	RS-anxious T2										–	0.60***	-0.11***
12	RS-angry T2											–	-0.17***
13	Age												–


*^∗^*p* < 0.05; ^∗∗^*p* < 0.01; ^∗∗∗^*p* < 0.001; minimum *N* = 1,134.*

### Differences between Participants with Depressive Symptoms and Controls

Two MANCOVAs testing for differences in the sensitivity subscales between participants with above average depressive symptoms and controls controlling for age and gender revealed a significant multivariate group effect both at T1 [*F*(5,1456) = 7.685, *p* < 0.001, $\eta_{p}^{2}$ = 0.026; *n*_depressive_ = 49, *n*_controls_ = 1,415] and at T2 [*F*(5,1273) = 7.342, *p* < 0.001, $\eta_{p}^{2}$ = 0.035; *n*_depressive_ = 36, *n*_controls_ = 1,245]. Follow-up analyses using ANCOVAs showed that on subscale level, participants from the depressive symptoms groups reported higher anxious and angry RS and victim JS at T1 and T2. There were no differences in observer and perpetrator JS (**Table 1** for detailed results). Given large differences in sample sizes, we repeated the analyses using non-parametric *U-*tests yielding identical results except for additional significant differences in observer JS at T1 (*U* = 28244.5, *p* = 0.023) and T2 (*U* = 15246.5, *p* < 0.001). Further analyses with a matched control group that was identical in T1 age, gender, and attended class at T1 (*p* = 1.00, respectively) also corroborated these results. Hence, mostly in line with Hypothesis 1, participants with above average depressive symptoms showed higher anxious and angry RS as well as higher victim JS and partially higher observer JS. There were no differences in perpetrator JS.

### Long-Term Mutual Influences of Sensitivities and Depressive Symptoms

**FIGURE 1:**
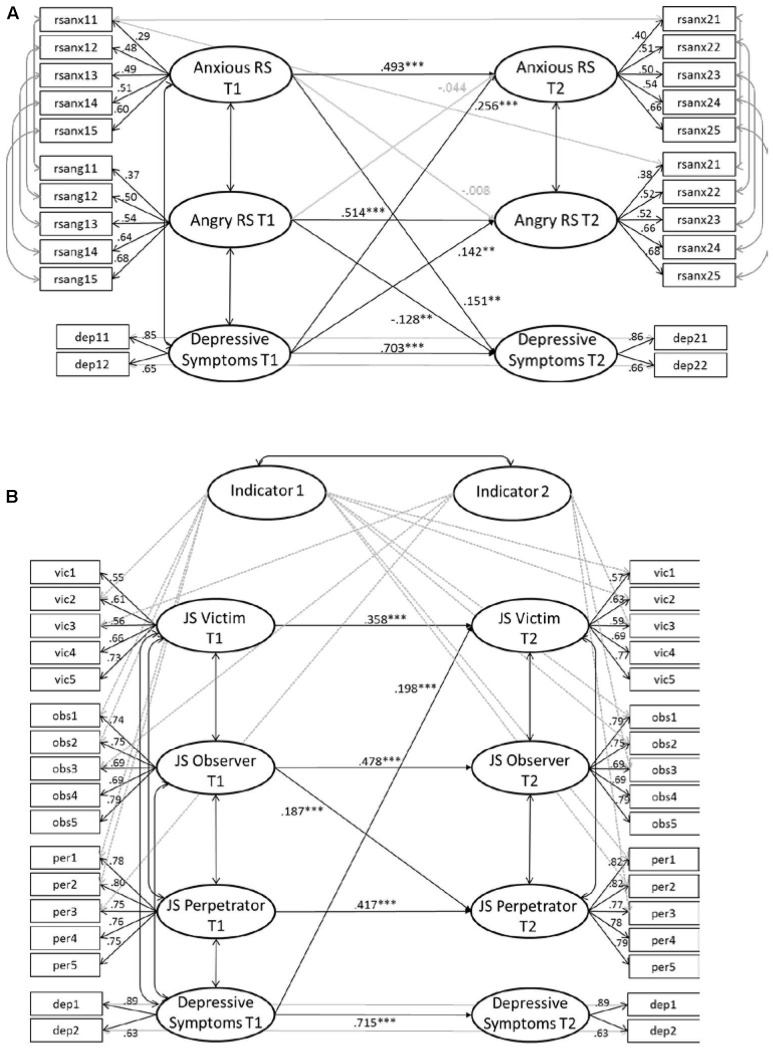
Cross-lagged models with RS **(A)**, JS **(B)**, and depressive symptoms assuming strong measurement invariance. RS: Error terms of the accordant items allowed to correlate (example displayed). JS: Error terms of the remaining two items of the JS subscales allowed to correlate (not displayed). Significant pathways and standardized model results displayed.

We examined the mutual long-term influences of RS and JS and depressive symptoms via latent cross-lagged models. We replaced missing data via the Full Information Maximum Likelihood procedure using M*plus* 7 (Muthén and Muthén, 1998–2012). We assumed strong measurement invariance for all measures between the two points of measurement because comparisons with cut-off criteria for absolute fit indices indicated good model fits (Hu and Bentler, 1998; Schermelleh-Engel et al., 2003) and because there were no decreases in the CFI exceeding 0.01 in the models assuming strong measurement invariance as compared to the models assuming weak measurement invariance (Cheung and Rensvold, 2002). Where applicable we also assumed strong measurement invariance between groups (see below). We computed two separate models to account for the singular mutual long-term influences of RS and JS and depressive symptoms. This proceeding allowed us to better compare the results on the links between RS and depressive symptoms in the present study to those from other studies (McCarty et al., 2007; Marston et al., 2010; McDonald et al., 2010; Zimmer-Gembeck et al., 2016). Latent factors of RS were indicated by five items per anxious and angry RS. Correlations of error terms of respective items between subscales and points of measurement were allowed and estimated (**Figure 1A**). Latent factors of JS were indicated by five items per scale. Two methods factors accounted for similar item wordings between JS subscales and points of measurement. Factor loadings of accordant T1 and T2 indicators on the methods factors were constrained to be equal. Correlations of error terms of the remaining two items of the JS scales with similar wordings were allowed and estimated (**Figure 1B**). Latent factors of depressive symptoms were indicated by the sum scores of the two depressive symptoms subscales. Correlations of error terms between the accordant T1 and T2 indicators were allowed and estimated. All indicators showed significant loadings on their latent factors. All T1 predictors were allowed to correlate. Correlations of T2 error terms between the sensitivity subscales were allowed and estimated. Correlations of error terms of T2 depressive symptoms and T2 sensitivity measures were restrained to zero. Given pronounced gender and age differences as indicated by the MANCOVA results, we restrained from controlling for these variables in the initial structural equation models and examined the moderating role of gender and age in separate models.

#### Rejection Sensitivity and Depressive Symptoms

A Confirmatory Factor Analysis (CFA) including T1 and T2 anxious and angry RS and depressive symptoms with factors correlated as described above confirmed the intended factor structure (χ^2^ = 864.610, *df* = 233, *p* < 0.001, RMSEA = 0.040, CFI = 0.950, SRMR = 0.069). The cross-lagged path model explained the data well (χ^2^ = 719.582, *df* = 233, *p* < 0.001, RMSEA = 0.035, CFI = 0.961, SRMR = 0.048, *N* = 1,665). All factors showed significant auto-regressions. Partially in line with Hypothesis 2a, higher T1 anxious RS predicted more T2 depressive symptoms: participants who feared and overreacted to rejection at first measurement tended to report more depressive symptoms 1–2 years later. Contrasting Hypothesis 2a, higher angry RS predicted *lower* T2 depressive symptoms: participants who angrily expected and overreacted to rejection tended to report less depressive symptoms later on. Supporting Hypothesis 3a, T1 depressive symptoms predicted higher T2 anxious and angry RS: participants showing a higher number of depressive symptoms at first measurement more strongly anxiously and angrily expected rejection 1–2 years later.

#### Justice Sensitivity and Depressive Symptoms

A CFA including all T1 and T2 JS and depressive symptoms measures with factors correlated as described above confirmed the intended factor structure (χ^2^ = 1492.429, *df* = 488, *p* < 0.001, RMSEA = 0.035, CFI = 0.959, SRMR = 0.053). The cross-lagged path model explained the data well (χ^2^ = 1409.324, *df* = 488, *p* < 0.001, RMSEA = 0.034, CFI = 0.963, SRMR = 0.041, *N* = 1,665). All factors showed significant auto-regressions. Contrasting Hypothesis 2b, none of the T1 JS scales predicted T2 depressive symptoms: JS did not promote depressive symptoms in the long run. Supporting Hypothesis 3b, more T1 depressive symptoms predicted higher T2 victim JS: Children and adolescents with many depressive symptoms at first measurement reported more anger and rumination in response to injustice to their own disadvantage 1–2 years later. Contrasting Hypothesis 3b, T1 depressive symptoms did not predict T2 perpetrator JS or T2 observer JS. Thus, participants with a large number of depressive symptoms at first measurement did not report stronger affective or cognitive responses to injustice toward others 1–2 years later.

#### Age and Gender Differences

In order to examine the moderating role of gender and age, we computed two multi-group cross-lagged models per sensitivity measure, allowing us to separately consider the influences of these two variables. Regarding gender, the model including RS with path weights allowed to vary between groups showed a good fit with the data (χ^2^ = 1078.921, *df* = 494, *p* < 0.001, RMSEA = 0.038, CFI = 0.954, SRMR = 0.055, *N* = 1,665). It had a significantly better fit than the model with path weights constrained to be equal between boys and girls (χ^2^ = 1137.305, *df* = 516, *p* < 0.000, RMSEA = 0.038, CFI = 0.951, SRMR = 0.056, *N* = 1,665; Δχ^2^ = 58.384, Δ*df* = 22, Δ*p* < 0.001). Wald tests did not reveal significant differences in path coefficients between groups (**Table 3** for details). Hence, contrasting Hypothesis 5, RS and depressive symptoms were similarly related in girls and in boys.

**Table 3:** Path weights from the multi-group models controlling for differential links of the rejection sensitivity and the justice sensitivity scales with depressive symptoms as moderated by gender and age, respectively.

	Boys	Girls	Children	Adolescents
T1 Anxious RS – T2 DS	0.021	0.108	0.170*	0.039
T1 Angry RS – T2 DS	-0.012	-0.047	-0.144*	-0.050
T1 DS – T2 Anxious RS	0.192*	0.274***	0.245***	0.213*
T1 DS – T2 Angry RS	0.090	0.191**	0.131*	0.139
T1 JS Victim – T2 DS	0.080	-0.012	-0.089	0.099
T1 JS Observer – T2 DS	-0.096a	0.126+b	0.170a	-0.116b
T1 JS Perpetrator – T2 DS	0.001	-0.047	-0.050	0.104
T1 DS – T2 JS Victim	0.161**	0.201***	0.224***	0.105***
T1 DS – T2 JS Observer	0.062	0.017	0.064	0.038
T1 DS – T2 JS Perpetrator	-0.076	-0.037	-0.058	0.072


*Superscripts ^a,b^*p* < 0.05 significant differences between gender or age group according to χ^2^-difference test; only paths linking sensitivity subscales with depressive symptoms displayed.*

Regarding the moderating role of gender in the link of JS and depressive symptoms, the model with path weights allowed to vary between boys and girls fit the data well (χ^2^ = 2043.428, *df* = 1007, *p* < 0.001, RMSEA = 0.035, CFI = 0.956, SRMR = 0.045, *N* = 1,665) and significantly better than the more restrictive model with path weights constrained to be equal between groups (χ^2^ = 2166.021, *df* = 1058, *p* < 0.001, RMSEA = 0.035, CFI = 0.953, SRMR = 0.056, *N* = 1,665; Δχ^2^ = 122.593, Δ*df* = 51, Δ*p* < 0.000). However, contrasting Hypothesis 5, only T1 observer JS predicted less depressive symptoms in boys and more depressive symptoms in girls 1–2 years later.

Concerning the moderating role of age in the link between RS and depressive symptoms, the multi-group model with path weights allowed to vary between children and adolescents showed a good fit with the data (χ^2^ = 1062.943, *df* = 481, *p* < 0.001, RMSEA = 0.040, CFI = 0.951, SRMR = 0.058, *N* = 1,665) and a significantly better fit than the model with path weights constrained to be equal across groups (χ^2^ = 1337.432, *df* = 516, *p* < 0.001, RMSEA = 0.046, CFI = 0.931, SRMR = 0.078, *N* = 1,665; Δχ^2^ = 274.489, Δ*df* = 35, Δ*p* < 0.000). Inspections of path coefficients indicated closer links of RS and depressive symptoms in children than in adolescents, but supporting Hypothesis 6, path coefficients did not differ significantly between age groups according to Wald tests.

Regarding the moderating role of age in the links of the JS subscales and depressive symptoms, the multi-group model with path weights allowed to vary between children and adolescents showed a good fit with the data (χ^2^ = 2136.952, *df* = 1007, *p* < 0.001, RMSEA = 0.039, CFI = 0.951, SRMR = 0.052, *N* = 1,665) and a significantly better fit than the model with path weights constrained to be equal across groups (χ^2^ = 2246.836, *df* = 1058, *p* < 0.001, RMSEA = 0.039, CFI = 0.949, SRMR = 0.057, *N* = 1,665; Δχ^2^ = 109.884, Δ*df* = 51, Δ*p* < 0.000). In line with Hypothesis 6, differences between path coefficients were minimal. Wald tests only revealed significant differences in path coefficients between children and adolescents of T1 observer JS to T2 depressive symptoms: Higher T1 observer JS predicted more depressive symptoms in children and less depressive symptoms in adolescents at T2.

#### Changes in Depressive Symptoms

**FIGURE 2:**
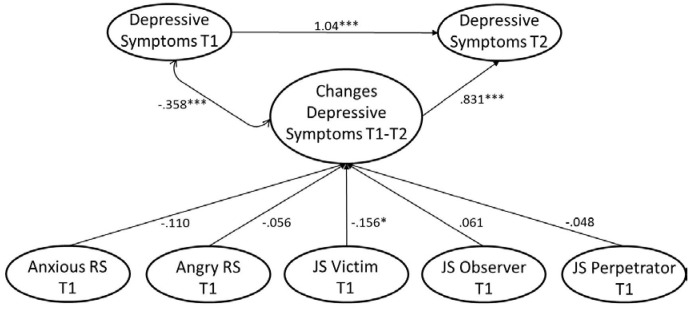
Latent-change model with Tl sensitivity subscales predicting changes in depressive symptoms between Tl and T2 assuming strong measurement invariance in depressive symptoms and controlled for gender and age. Measurement model for sensitivity subscales as in **Figure 1**. Additional indicator factor for second indicators of depressive symptoms at Tl and T2. Factor loadings on the indicator factor constrained to be equal. All predictors allowed to correlate. Standardized model results displayed.

Following our reasoning, the sensitivities should not only predict rank-order changes in depressive symptoms, but also changes in the mean number of depressive symptoms between T1 and T2. More precisely, the sensitivities should add to a stabilization of depressive symptoms. In order to examine this question, we computed a latent-change model. In this model, we considered the simultaneous influences of RS and JS, controlled for gender and age, and considered only those participants who took part in our study at both points of measurement (**Figure 2**). The sensitivity factors were modeled as described above. As in the manifest data, mean differences indicated a general decrease in depressive symptoms from T1 to T2. Persons with a higher number of depressive symptoms at T1 tended to show smaller mean level changes—particularly in terms of a decline—in depressive symptoms as indicated by the significant negative pathway from T1 depressive symptoms to the difference score. Higher victim JS predicted lower mean level changes from T1 to T2, indicating that participants who reported anger, strain, and rumination in response to injustice, were prone to smaller changes, that is declines, in the number of depressive symptoms (χ^2^ = 816.369, *df* = 385, *p* < 0.001, RMSEA = 0.032, CFI = 0.962, SRMR = 0.052, *N* = 1,093). Hence, higher victim JS apparently promotes the stabilization of depressive symptoms.

## Discussion

The present study examined the links of rejection and justice sensitivity as examples for traits that guide individual’s perceptions and responses to social cues with depressive symptoms in children and adolescents. To reach this aim, we investigated the mutual long-term influence of RS and JS and depressive symptoms via latent cross-lagged and latent-change models in a large sample of German children and adolescents. In line with previous research (Marston et al., 2010) and our assumptions, anxious RS was a cause and consequence of depressive symptoms. Contrary to our assumptions, higher angry RS predicted less depressive symptoms. Also contrasting our assumptions, JS did not add to the prediction of depressive symptoms. Instead, more depressive symptoms at first measurement predicted more intense adverse reactions to perceived injustice to one’s own disadvantage (victim sensitivity) at T2. When considered simultaneously with the other sensitivity measures, however, victim JS was the sole predictor of smaller declines in depressive symptoms over the course of 1–2 years, apparently adding to the stability of depressive symptoms in those participants remaining in our study.

### Mutual Links of Rejection and Justice Sensitivity and Depressive Symptoms

Prevalence rates for above average depressive symptoms in the present sample resembled previous findings in this age range (Esser et al., 1990; Ihle et al., 2000). Also in line with previous findings (Angold et al., 1998; Wittchen et al., 1998; Groen and Petermann, 2005), girls reported significantly more depressive symptoms than boys. However, contrary to previous findings (Wittchen et al., 1998; Ihle et al., 2000; Preiß and Remschmidt, 2007), the average number of depressive symptoms and prevalence rates decreased between T1 and T2. Drop-out analyses did not indicate a systematic drop-out of participants with initially high levels of depression, but a systematic drop-out of older participants who may have been more prone to develop a larger amount of depressive symptoms between the two points of measurement. Furthermore, a decline in the average number of depressive symptoms between T1 and T2 may also be indicative of a trend of regression to the mean in those with higher levels of depressive symptoms at first measurement in particular.

In line with previous research on the links of RS and depressive symptoms (Marston et al., 2010), anxious RS was a cause *and* a consequence of depressive symptoms. Hence, depressive symptoms and expectations of as well as angry and anxious affective reactions to rejection may mutually enhance one another in the long run, likely resulting in a vicious circle of fearing rejection and depressive reactions. This may work to further impair social relationships presumably resulting in more alleged or actual rejection. Although anxious RS seems to cause depressive symptoms, it did not add to their stabilization, which was better explained by victim JS.

Also in line with previous research (McDonald et al., 2010), anxious *and* angry RS showed positive zero-order correlations with depressive symptoms. However, when the effects of anxious RS were controlled for, the tendency to angrily expect rejection predicted a *decrease* in depressive symptoms. This indicates a suppressor effect most likely due to the control of the cognitive facet of RS, that is, the expectation of rejection that influences both anxious and angry RS. Thus, contrary to our expectations, angry expectations of rejection apparently prevent from developing further symptoms of depression. Instead, individuals high in angry RS may tend to develop externalizing problem behavior (Zimmer-Gembeck and Nesdale, 2013). Hence, those high in angry RS may tend to direct negative feelings and behavior against others instead of against themselves. A high correlation of the two RS subscales (t3: *r* = 0.65), indicating some level of multicollinearity, also has to be kept in mind when interpreting these findings.

Depressive symptoms at first measurement, however, increased the risk for both anxious and angry expectations 1–2 years later. This may be due to the fact that depression promotes negative perceptions of social interactions in line with the cognitive scar hypothesis (Rohde et al., 1990; see below) or that depressive symptoms add to a factual deterioration of social relationships irrespective of the emotion primarily associated with these expectations (concern or anger). However, in line with our reasoning, links of anxious RS with depressive symptoms were somewhat more pronounced than those of angry RS, most likely due to the fact that concern and sadness as associated with anxious RS are more closely related to depressive symptoms than anger as associated with angry RS.

Concerning JS, in line with previous research (Lovaš and Wolt, 2002; Bondü and Esser, 2015) all JS subscales were significantly positively correlated with depressive symptoms. However, only victim JS showed long-term relations with depressive symptoms, but only in the latent change model. Contrasting our expectations, none of the JS subscales predicted depressive symptoms 1–2 years later in the latent cross-lagged model, indicating that (even victim) JS does not have as strong depressogenic effects as anxious RS. This may indicate that even high victim JS does not necessarily impair social relationships in terms of a self-fulfilling prophecy as has been shown with regard to RS (Ayduk et al., 2001).

Depressive symptoms at first measurement, however, predicted higher victim JS 1–2 years later. This is in line with research that has related victim JS to rumination, anger, sadness, helplessness, and social withdrawal (Bondü et al., in preparation; Schmitt et al., 1995). Initial depressive symptoms apparently add to the tendency to intensely respond to injustice to one’s own disadvantage for example via anger, sadness, stress, and rumination. Also, even though victim JS did not add to the prediction of depressive symptoms, it apparently added to the stabilization of these symptoms if they were already present, thus, hindered a decline in the number of depressive symptoms in childhood and adolescence. This effect exceeded that of anxious RS, indicating that injustice to one’s own disadvantage is similarly aversive as rejection. Hence, negative affect, strain, and rumination associated with victim JS may stabilize depressive symptoms. Thus, victim JS may rather be a maintaining factor than a cause of depression.

The present study is the first to show that JS not only relates to externalizing, but also to internalizing problem behavior. Particularly victim JS is linked to a broad range of maladaptive behaviors (Bondü and Elsner, 2015; Bondü and Esser, 2015), indicating that the adverse affective and cognitive responses to injustice associated with victim JS predispose or relate to a broad range of mental health problems. Hence, victim JS seems an important factor in developmental psychopathology and should gain more attention by future research.

Overall, effects from depressive symptoms on RS and JS were somewhat more pronounced and consistent than the opposite effects. This may be due to higher stability rates of the number of depressive symptoms than of the sensitivity measures, leaving more variance to be explained in the sensitivities measures in the latent models. Hence, although RS and JS are traits, they may be influenced by internal processes and interpersonal experiences, which may also explain some of their variability (Bondü and Elsner, 2015).

### Potential Ways of Effect

To date there is little knowledge about the mechanism and processes that may explain the effects from RS on depressive symptoms and from depressive symptoms on RS and JS. Several ways of effect can be assumed and future research may want to examine them.

#### Sensitivity to Depression

The sensitivity measures may promote the emergence (anxious RS) or the stabilization (victim JS) of depressive symptoms in a number of ways:

(1) RS and JS may work in a fashion similar to dysfunctional thoughts (Bondü and Esser, 2015): they may promote negative social expectations and attributions as well as a focus on negative aspects of social interactions as described by the Interpersonal Life-Stress Model of depression (Rudolph et al., 2000). Furthermore, RS and JS may also indirectly add to the impairment of social relationships via decreases in social competencies (Marston et al., 2010). This is also likely result in a decrease of social support in crisis, thereby impeding effects that may otherwise buffer depression.
(2) Negative expectations, attributions, and interactions after perceived rejection or injustice may evoke feelings of worthlessness, loneliness, despair, and hopelessness and impair self-esteem (Ayduk et al., 2001; Bondü and Esser, 2015). These factors are closely related to the development of depression and/or constitute depressive symptoms themselves (Abramson et al., 1989; Zimmer-Gembeck et al., 2014).
(3) The strain associated with perceptions of rejection or injustice may work as a stressor (i.e., daily hassles) promoting depression in line with the diathesis-stress model (Lewinsohn et al., 2001).
(4) Regarding RS, research has found worry to be an influential moderator of the aggression-depression link in physically aggressive girls (Blain-Arcaro and Vaillancourt, 2016). Hence, worry and expectations of rejection as associated with anxious RS may work to evoke depressive symptoms in girls in particular.
(5) Regarding JS, victim JS is related to sadness, self-pity, helplessness, fear of victimization, and social withdrawal (Bondü et al., in preparation). These factors closely resemble antecedents and symptoms of depression and are similar to responses connected with anxious RS. Hence, similar to the distinction between anxious and angry RS, there may be some victim-sensitive individuals that tend to respond with sadness and withdrawal to experiences of injustice, whereas others may tend to respond by anger and aggression.
(6) Rejection may have negative and enduring physical consequences such as impeding hormonal balance that may impair physical and mental health in the long run (Slavich et al., 2010).

#### Depression to Sensitivity

There are also processes via which depressive symptoms may promote RS and JS:

(1) In line with the Interpersonal Life-Stress model for depression (Rudolph et al., 2000), overly negative interpretations of social cues as associated with RS and JS may result in a vicious circle of misinterpretations of others’ behavior, adverse responses toward this behavior (Downey and Feldman, 1996; McCarty et al., 2007), social conflicts, withdrawal, depressive symptoms, negative expectations, and negative interpretations of social interactions, finally resulting in more negative social interactions. Given that negative social interactions may lead to a further sensitization toward negative social cues in the long run, this process may result in an increase in JS and RS as well.
(2) Similarly, Chango et al. (2012) have argued that rejection-sensitive individuals are inclined to develop negative believes about themselves and tend to use global, stable, and internal attributions to explain negative experiences. Thus, in line with the cognitive scar hypothesis of depression (Rohde et al., 1990) suggesting that there are long-term changes in cognitive style caused by depressive episodes, a pessimistic explanatory style may make more sensitive to rejection and injustice, even after depression subsides (McCarty et al., 2007). Therefore, however, it may predispose to future depressive symptoms and episodes in the long run.
(3) Finally, opioid activity easing physical and social pain has been found to be impaired in depression, impeding recovery from adverse social experiences and enjoying positive social interactions (Hsu et al., 2015). This process may also add to a further sensitization and increases in RS and JS in the long run.

### Age and Gender Differences

Both gender and age differences in the links of the sensitivity measures and depressive symptoms were negligible. Thus, despite well-known gender- and age-differences in RS, JS, and depressive symptoms (e.g., Bondü and Elsner, 2015) the relations between these constructs were similar across groups. Findings on opposed relations of observer sensitivity with depressive symptoms 1–2 years later in girls and boys resemble gender differences in cross-sectional links of victim and observer JS with aggressive behavior in adults (Bondü and Richter, 2016). They indicate that males and females cope differently with victim and observer sensitivity. Possibly, victim JS may predispose to externalizing behavior and observer JS may predispose to internalizing behavior in females, whereas in males, victim JS may predispose to internalizing behavior and observer JS may predisposes to externalizing behavior. More research, however, is needed, to test this assumption.

### Limitations and Outlook

The strengths of the present study include the large sample size, the use of multiple sensitivity measures, the use of longitudinal data, the consideration of mutual influences of the sensitivity measures and depressive symptoms, relating JS to internalizing problem behavior, and the examination of the moderator effects. Limitations include the low internal consistencies of the RS measures (see Zimmer-Gembeck and Nesdale, 2013, for similarly low internal consistencies of a short measure of RS) as well as indications of a systematic drop-out of older participants and those with lower levels of perpetrator sensitivity, both inclined to limit the generalizability of our findings. In addition, high interrelations of the two RS measures may indicate multicollinearity and may make the interpretation of the results difficult. Because the sequence of measures was held constant for all participants (RS, JS, depressive symptoms), we were not able to control for sequence effects. Also, we did not control for reading skills or whether items were read aloud to participants or not. In general, effects tended to be small. This was also reflected in the findings that there were significant links for example of anxious RS and depressive symptoms in the total group, but not in any of the subgroups. This indicates that the large sample size was required to identify significant effects. Although effects in the group comparisons were only small, the findings of the present study provide more detailed insights into why the interpersonal context is an important source for stress that may promote depressive symptoms, why this stress may affect some individuals more strongly than others, and how it works long-term. We chose 1.5 standard deviations as the cut-off criterion for the group with above average depressive symptoms. Using this criterion, the prevalence rates reported in the present study resembled those of previous research (Esser et al., 1990; Ihle et al., 2000). Furthermore, results were similar to those with a 2.0 *SD* criterion. Other criteria, however, might have been chosen. Finally, we have outlined a number of ways via which depressive symptoms may affect the sensitivities and via which RS and JS may affect depressive symptoms. However, we were not able to test these ways of effect in the present study due to a lack of accordant measures.

Thus, future research should examine the ways of effect from RS on depressive symptoms and from depressive symptoms on RS and victim JS. The effects of other important cognitive and emotional risk factors for depression (e.g., dysfunctional thoughts, negative attributions, hopelessness, helplessness) should be controlled for. We have suggested that RS and JS may mediate the link between negative social experiences and depressive symptoms. Future research using three points of measurement could examine this link in more detail. Also, it might be tested whether moderation or mediation models seem more appropriate to explain the effects when considering their interaction with further potential risk factors, such as social stressors. Given that depressive symptoms may first get obvious during childhood, the links of RS and JS with depressive symptoms may be investigated at earlier stages of development. Finally, future research should examine the links between JS and other forms of internalizing problems and also include other rating-sources.

Our study has important theoretical and practical implications for the research on and the prevention and treatment of depressive symptoms: Our findings indicate that interpersonal sensitivity factors such as RS and JS (anxious RS and victim JS in particular) may promote the genesis and/or stabilization of depressive symptoms in children and adolescence. Hence, these factors deserve more attention in theoretical frameworks. One way to simultaneously consider cognitive vulnerability factors, interpersonal stressors, and sensitivities toward adverse social cues may be to combine them into a cognitive-interpersonal theory of depression, focusing on interaction-related cognitions and feelings in particular.

The treatment of depressive symptoms has already focused on cognitive-behavioral factors and applied cognitive techniques and fostered social competencies (Reinecke and Ginsburg, 2008). Our results further corroborate this approach and suggest the importance of fostering social competencies and social relations in order to break the potentially self-perpetuating circle of negative social interactions and more depressive symptoms at early stages of development. It also seems important to communicate the potential negative effects that own behavior may have on others and on their responses in terms of psychoeducation.

## Author Contributions

All authors listed have made a substantial, direct and intellectual contribution to the work, and approved it for publication.

## Conflict of Interest Statement

The authors declare that the research was conducted in the absence of any commercial or financial relationships that could be construed as a potential conflict of interest. The reviewer MM and handling Editor declared their shared affiliation.
